# Complexes of Zinc With Picolinic and Aspartic Acids Inactivate Free Varicella-Zoster Virions

**DOI:** 10.1155/MBD.1996.11

**Published:** 1996

**Authors:** S. Shishkov, T. Varadinova, P. Bontchev, C. Nachev, E. Michailova

**Affiliations:** 1 Laboratory of Virology Faculty of Biology University of Sofia 8 Dragan Tzankov Blvd. Sofia 1421 Bulgaria; 2 Laboratory of Virology Faculty of Chemistry University of Sofia Bulgaria; 3 Laboratory of Virology Higher Medical Institute Sofia Bulgaria

## Abstract

Zn(II) picolinate and aspartate, Zn(pic)_2_ and Zn(asp)_2_, have been shown to inhibit key steps of the replication of HSV-1. In the present study we describe the effect of Zn(pic)_2_ and Zn(asp)_2_ on the replication of VZV and on the infectivity of free virions. The experiments are done using BHK-21 cells, a clinical isolate of VZV and Zn-complexes in concentration of 10 *μ*M. When Zn-complexes are present during the whole period of infection, the yield of infectious virus progeny decreases up to 98%. The infectivity of VZV is completely restored after the removal of zinc. The virucidal effect is manifested at the 2nd h of contact, when 90% of the virions are inactivated. The results show that both Zn(pic)_2_ and Zn(asp)_2_ specifically inactivate free VZV virions with no effect on viral replication.


### COMPLEXES OF ZINC WITH PICOLINIC AND ASPARTIC ACIDS INACTIVATE FREE VARICELLA-ZOSTER VIRIONS

S. Shishkov[1], T. Varadinova*[1], P. Bontchev[2], C. Nachev[3] and E. Michailova[1]

[1] Laboratory of Virology, Faculty of Biology, University of Sofia, 8 Dragan Tzankov Blvd., 1421 Sofia, Bulgaria

[2] Faculty of Chemistry, Univesity of Sofia, Bulgaria

[3] Higher Medical Institute, Sofia. Bulgaria

**Abstract**

Zn(II) picolinate and aspartate, $Zn(pic)_2$ and $Zn(asp)_2$, have been shown to inhibit key steps of the replication of HSV-1. In the present study we describe the effect of $Zn(pic)_2$ and $Zn(asp)_2$ on the replication of VZV and on the infectivity of free virions. The experiments are done using BHK-21 cells, a clinical isolate of VZV and Zn-complexes in concentration of 10 $\mu$M. When Zn-complexes are present during the whole period of infection, the yield of infectious virus progeny decreases up to 98%. The infectivity of VZV is completely restored after the removal of zinc. The virucidal effect is manifested at the 2nd h of contact, when 90% of the virions are inactivated. The results show that both $Zn(pic)_2$ and $Zn(asp)_2$ specifically inactivate free VZV virions with no effect on viral replication.

Key words: Varicella-zoster virus, zinc, replication, inhibition, inactivation.

### Introduction

Varicella-zoster virus (VZV) belongs to the genus *Varicellovirus* of the subfamily *Alphaherpesvirinae*, family *Herpesviridae* (1). Members of the same subfamily are also Herpes simplex viruses types 1 and 2 (2). Despite structural and antigenic relationships, significant differences in biological properties between VZV and Herpes simplex virus (HSV) are evident. Thus, VZV is mainly cell associated (1, 3, 4). The virus uses a noncanonical transcription start and stop sites. The fully assembled nucleocapsids mature by two step envelopment - during the transport through inner nuclear membrane and during the migration into vacuoles. Within these vacoules, mature virions migrate to the cell periphery and are released by exocytosis (1, 5).

Because of the unique biological properties, the problems of therapy and control of VZV infection are still open. The current treatment of VZV infection for humans is based on Acyclovir (6). Despite the modestly shortened duration of varicella and zoster symptoms and the prevention of the spread of zoster to the eye in normal individuals, the existence of Acyclovir resistant VZV strains have been reported (1, 3, 7, 8). Other agents used in therapy of VZV infection are Vidarabine and alpha-Interferon (9, 10).

Our previously published data have shown that $Zn(pic)_2$ and $Zn(asp)_2$ inhibit key steps of HSV-replication (11, 12). However, the effect of these Zn-complexes on VZV infection *in vitro* is also of interest.

### Materials and Methods

***Varicella-zoster virus and cells.*** A clinical isolate of VZV adapted for replication *in vitro* and BHK-21 cell line were used. The viral stock was obtained from BHK-21 cells cultured for 72 h at 37 °C and then frozen and thawed.

***Infectious virus titre.*** Tube cells from suspension in amount of 0.9 ml were infected with 0.1 ml of VZV stock in ten-fold dilutions. After culturing at 37 °C for 72 h the infectious virus titre was determined by Reed and Muench (13).

***Effect of Zn-complexes on the replication of VZV.*** Simultaneously, 0.1 ml of media modified with 10 $\mu$M or 1 $\mu$M of each Zn-complex and 0.1 ml of VZV stock in various dilutions were added to 0.8 ml of cells from suspension. One set of infected cells served as untreated control.

The effect on VZV replication was determined by reduction of infectious virus titres as compared to that from untreated control at the 72nd h after culturing at 37 °C.

***Reversibility of the inhibitory effect.*** Samples from the above experiments containing 1000 pfu/0.1 ml of VZV and an appropriate concentration of each Zn-complex, as well as that from untreated controls, were frozen, thawed and diluted ten-fold. BHK-21 cells suspended in nonmodified medium were infected with appropriate dilution from each sample. The reversibility of the effect of Zn-complexes on the replication of VZV was determined by the restoration of viral infectivity as compared to that of the untreated control.

***Effect of Zn-complexes on extracellular (free) VZV virions (virucidal effect).*** Equal volumes of VZV stock containing 100 pfu/ml and media modified with 10 $\mu$M of appropriate Zn-complex were incubated at 37 °C for 15 min, 30 min, 1 h, 2 h, 4 h and 12 h. Each sample was diluted ten-fold and 0.9 ml suspended cells were infected with 0.1 ml of each dilution. The virucidal effect was determined at the 72nd h by reduction of infectious virus titres as compared to that of the viral control - equal volumes of VZV stock and unmodified medium incubated as described above.

### Results

The data presented in Table 1 show that $Zn(pic)_2$, as well as $Zn(asp)_2$, in a concentration of 10 $\mu$M, but not of 1 $\mu$M, inhibit the replication of VZV in BHK-21 cells by 98% and 90% respectively, as compared to the untreated viral control.

The inhibitory effect is reversible (Table 1). Thus, after treatment with $Zn(pic)_2$, the infectivity of VZV is completely restored while, after influence with $Zn(asp)_2$, 73% of the viral progeny reversed their infectious activity.

Table 1

Effect of $Zn(pic)_2$ and $Zn(asp)_2$ on the replication of VZV and the reversibility of the action

| Zn-complex | Consentration in $\mu$M | Effect on the replication | | Reversibility of the action | |
|---|---|---|---|---|---|
| $Zn(pic)_2$ | 10 | 6.2[a] | 98[b] | 3.6[a] | 0[b] |
| | 1 | 7.8 | 0 | n.d. | |
| $Zn(asp)_2$ | 10 | 6.9 | 90 | 3.5 | 27 |
| | 1 | 7.9 | 0 | n.d. | |
| VZV control | | 7.9 | | 3.7 | |

[a] lg pfu/0.1 ml; [b] Inhibition in %; n.d. - not done

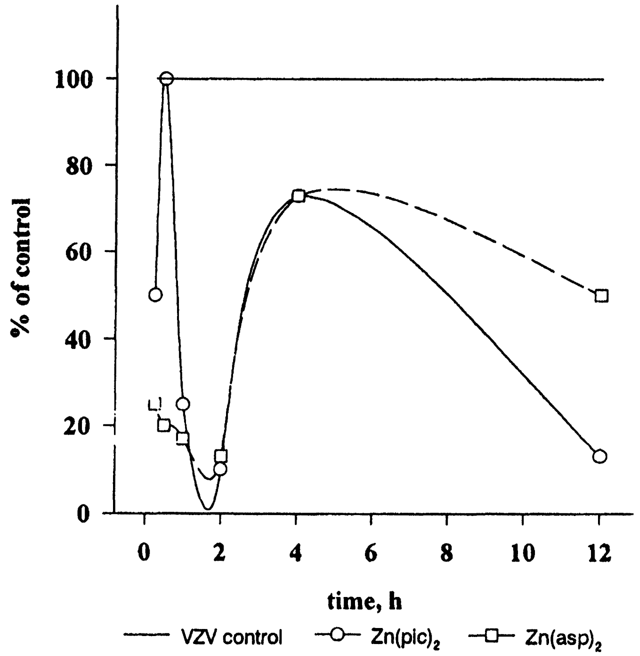

Fig. 1: Virucidal effect of $Zn(pic)_2$ and $Zn(asp)_2$

In order to clarify why the replication of VZV was inhibited in the presence of Zn-complexes and is completely restored immediately after removing zinc, we studied the effect of these complexes on extracellular virions.

Table 2
Effect of $Zn(pic)_2$ and $Zn(asp)_2$ on extracellular VZV virion - virucidal effect*

| Zn-complex** | Duration of the contact | | | | | |
|---|---|---|---|---|---|---|
| | 15min | 30min | 1h | 2h | 4h | 12h |
| $Zn(pic)_2$ | 7.6 | 6.9 | 5.7 | 5.0 | 5.3 | 4.1 |
| $Zn(asp)_2$ | 7.3 | 6.2 | 5.5 | 5.1 | 5.3 | 4.7 |
| VZV control | 7.9 | 6.9 | 6.3 | 6.0 | 5.5 | 5.0 |

*data are presented as lg pfu/0.1 ml

**each complex is applied in a concentration of 10 μM measured by Zn(II)

The results presented in table 2 and fig. 1 show two sets of data. First - the specific dynamics of the virucydal effect of Zn-complexes manifested between the 2nd h and 12th h of the contact with free VZV. Thus, at the 2nd and 12th h after contact, 90% and 87% respectively of VZV virions are inactivated. In contrast, at the 4th h only 17% of the free VZV are inactivated. Second - the virucidal effect of Zn-complexes during the first two hours of contact with VZV virions depends on the ligands of Zn(II). Thus, $Zn(asp)_2$ affects free VZV soon after the contact and at the 15th min 75% of the virions are already inactivated. The effect increases with the prolongation of the contact up to the 2nd h. Conversely, $Zn(pic)_2$ inactivates free VZV 1 h after the contact with prolongation of the effect till the 2nd h.

**Discussion**

The data presented here show that when suspended BHK-21 cells are influenced with VZV and $Zn(pic)_2$ or $Zn(asp)_2$, viral replication is inhibited by 90-98%. According to the experimental protocol, two events proceed simultaneously on the plasma membrane - viral attachment and absorption of Zn-complex. During their movement from the medium to the cell surface Zn-complex and VZV particles can interact with each other. It is well known that the VZV envelope originates from cell membranes modified by viral glicoproteins (4, 5). That is why we suggest that the inhibition of VZV replication by Zn-complex is due to the specific kinetics of Zn(II) exposure onto membranes (12) rather than to the direct effect on viral replication into host cells. The following three sets of results are in accordance with this suggestion. Firstly, the complete restoration of VZV infectivity after removal of zinc (table 1). Secondly, a weak effect of 10 μM $Zn(pic)_2$ on the replication of $tk^+$ and $tk^-$ strains of VZV, obtained by Dr. J. Neits (data not published) from the team of Prof. E. De Clercq (Rega Institute for Medical Research, Katholieke Universiteit, Leuven). In these experiments $Zn(pic)_2$ has been added after VZV attachment. Thirdly, inactivation of free VZV virions on the 2nd h after contact with $Zn(pic)_2$ or $Zn(asp)_2$ (table 2, fig. 1).

In addition, the ligand of Zn(II) determines the duration and the degree of the inactivating effect of this ion on VZV virions. Thus, aspartic acid ensures quick and a prolonged (at least 2 h) virucidal effect of Zn(II). On the other hand, picolinic acid predetermines a brief and delayed effect on free virions which is manifestated between 1st and 2nd h.

The different effects of $Zn(pic)_2$ and $Zn(asp)_2$ on VZV and HSV replications are obviously due to the specific biological properties unique to both viruses.

**Acknowledgements:** This work is financially supported by grants X-15 and L-451 from the National Foundation for Science.

**References**

1. J.I.Cohen, S.E. Straus, W.T. Ruyechan, J.Hay. In: *Encyclopedia of Virology.* R.E. Webster, A. Granoff (eds.), Acad. Press, New York, 1994, **v.3**, 1514.
2. L. Aurelian. In: *Encyclopedia of Virology.* R.E. Webster, A. Granoff (eds.), Acad. Press, New York, 1994, **v.2**, 587.
3. S.E. Straus, I.M. Ostrove, G. Inchauspe, J.M. Felsez, A. Freifild, K.D. Kroen, M.H. Sawyer. *Ann. Intern. Med.* 1988, **108**, 221.
4. L. Gleb. In: *Virology.* B.N. Fields et al. (eds.), Raven Press, N.Y., 1990, 2011.
5. J.M. Ostrove, *Adv. Virus Res.*, 1990, **38**, 45.
6. R. Snoeck, G. Andrei, E De Clercq. *Intern. J. Antimicrob. Agents,* 1994, **4**, 211.
7. K.J. Smith, C. Kahlter, C. Davis, W.D. James, H.G. Skelton, B. Angriff. *Arch. Dermatol.* 1991, **127**, 1069.
8. C.C. Linnemann, K.K. Biron, W.G. Hoppenjans, A.M. Solinger. *AIDS*, 1990, **4**, 577.
9. R.J. Whitley, M. Hilty, R. Hynes, Y. Brison, J.D. Connor, S.J. Soong, C.A. Alford. *J. Pediatr.* 1982, **101**, 125.
10. A.M. Arvin, J.H. Kushner, S. Foldman, R.L. Buchner, D. Itmond, T.C. Merigan. *N. Engl. J. Med.* 1982, **1**, 125.
11. T. Varadinova, I. Pavlov, D. Dimitrov, C. Nachev, P. Bontchev, B. Evtimova. *Compt. Rend. de l'Acad. Bulg. Sci.* 1990, **11**, 131.
12. T.L. Varadinova, P.R. Bontchev, C.K. Nachev, S.A. Shishkov, D. Strachilov, Z. Paskalev, A. Toutekova, M. Panteva. *J. Chemotherapy,* 1993, **5**, 1, 3.
13. L. Reed, H. Muench. *An. J. Hyg.*, 1938, **21**, 493.

**Received: March 2, 1995 - Accepted: April 19, 1995 - Received in revised camera-ready format: December 6, 1995**

